# Early social and ecological experience triggers divergent reproductive investment strategies in a cooperative breeder

**DOI:** 10.1038/s41598-020-67294-x

**Published:** 2020-06-26

**Authors:** Diogo F. Antunes, Barbara Taborsky

**Affiliations:** Institute of Ecology and Evolution, University of Bern, Bern, Switzerland

**Keywords:** Social evolution, Evolution

## Abstract

Unlike eusocial systems, which are characterized by reproductive division of labour, cooperative breeders were predicted not to exhibit any reproductive specialization early in life. Nevertheless, also cooperative breeders face a major life-history decision between dispersal and independent breeding vs staying as helper on the natal territory, which might affect their reproductive strategies. In the cooperatively-breeding cichlid *Neolamprologus pulcher* early-life social and predator experiences induce two behavioural types differing in later-life social and dispersal behaviour. We performed a long-term breeding experiment to test whether the two early-life behavioural types differ in their reproductive investment. We found that the early-dispersing type laid fewer and smaller eggs, and thus invested overall less in reproduction, compared to the philopatric type. Thus *N. pulcher* had specialised already shortly after birth for a dispersal and reproductive strategy, which is in sharp contrast to the proposition that reproductively totipotent cooperative breeders should avoid reproductive specialization before adulthood.

## Introduction

Cooperative animal societies range from eusocial species with a clear division in fecund queens and sterile workers^1^ to systems where subordinate group members help raising dominants’ offspring but remain fully fertile. Eusociality occurs predominantly in insect species, in which large breeding females are capable to produce far more offspring than the much smaller subordinates could do^2–4^. Insect queens have evolved morphological specializations allowing them to maximize reproductive investment as they do not incur the physiological constraints of internal gestation^4^. Contrary to eusocial insects, in cooperative breeding systems as occurring in many vertebrates, a dominant breeder pair producing all or most of the offspring is assisted by fully fecund subordinates, which delay natal dispersal and forgo own reproduction to help raising the dominants’ offspring (‘helpers’)^5,6^. In cooperative breeders an early specialization of reproductive strategies should nevertheless not be expected, because all subordinates can potentially become breeders themselves at some stage of their life^4^. Maintaining full flexibility of the reproductive strategy would enable subordinates to respond quickly to any opening opportunities for independent breeding^4^. Accordingly, there is no evidence for reproductive specialization from early life onwards in any cooperative breeder to date.

Many cooperative breeders face a major life history decision early in life, namely whether to delay dispersal into later adulthood and stay as subordinate brood care helper or to disperse soon after attaining sexual maturity and to breed independently^7–9^. This major life-history decision should influence an individual’s reproductive opportunities, and therefore it might affect major life history traits already well ahead of this major decision, such as age and size of sexual maturation, and the development of reproductive organs. Delaying dispersal and queueing for inheritance of a breeder position in the natal territory is often the safer option compared to dispersal. Philopatric individuals may benefit from group defence against predators^10,11^ or from sentinel duties taken up by group mates^12–14^. They may also benefit from parental nepotism when parents provide offspring access to resources in the natal territory not available outside the territory^15^. These benefits may render the natal territory “safe havens”, which enhance survival chances while queuing for a reproductive vacancy^12^. However, if conditions allow, benefits of dispersal may offset benefits of natal philopatry by an advanced onset of own, independent reproduction and by avoiding local competition with other group members. For instance, if in the social spider *Anelosimus jabaquara*, local food competition prior to egg laying is high, large females in good nutritional state disperse to avoid this competition, and they lay larger clutches, but with smaller eggs outside their natal territory, compared to philopatric females^16^.

In the cooperatively breeding cichlid fish *Neolamprologus pulcher*, a large breeder pair is assisted in raising its offspring by 1 to up to 20 smaller subordinate helpers, which engage in direct brood care (egg cleaning and fanning), and guarding and defence against predators. Groups are structured by a size-dependent social hierarchy. In a two-by-two factorial developmental experiment the early social and early predator experiences of *N. pulcher* had been manipulated in the laboratory, which revealed that these two environmental factors interactively shape the behavioural phenotype of individuals as well as their decision whether to stay as subordinate in a social group or to disperse early for own breeding^17^. The interaction between social and predator experiences resulted in a specialization into two behavioural types: Individuals raised during their first 2 months of life *with* older group members and *with* predator experience (+F/+P) and those raised *without* older group members and *without* predator experience (−F/−P) engaged more in helping behaviour later in life and dispersed from their group as soon as given the opportunity to do so. Instead, individuals raised *with* older group member and *without* predator experience (+F/−P) and those raised *without* older group member and *with* predator experience (−F/ + P) engaged more in submissive displays during later life and remained philopatric^17^.

We hypothesized that the early behavioural specialization of *N. pulcher* in early vs late dispersers affects their reproductive investment strategies, because in their natural habitat the timing of dispersal would influence the timing of the onset of reproduction. Specifically, we predicted that fish reared under environmental conditions that induced early dispersal (−F/−P and +F/+P)^17^ also had prepared for the immediate onset of reproduction after dispersal. Therefore they should (i) be able to reproduce more readily and at a faster rate, and (ii) possibly also invest more energy in reproduction relative to their body size, compared to philopatric individuals remaining as subordinate group members for an extended period of time (i.e., fish reared in +F/−P and –F/ + P conditions^17^).

To quantitatively compare the differences of reproductive investment strategies between individuals that received different early-life experiences, and to test if these experiences can lead to life-long, divergent reproductive investment, it requires a standardised breeding experiment with (i) rearing the experimental individuals under controlled early environmental conditions, (ii) then keeping them under identical conditions until reproduction and finally (iii) exposing them to breeding trials with experimentally assigned mating partners. To achieve this, we made use of the above-mentioned laboratory population of *N. pulcher*, in which early-life social and predator experiences had previously been manipulated in a two-by-two factorial experiment^17^. We used 4-years old adults that had been reared in four combinations of early-life environments as outlined above, that is, with parents and helper fish (+F) or without such older group members (−F) and with (+P) or without (−P) predator experience, paired them with partners from the same treatment combination, and then tracked their reproductive investment across three consecutive breeding events. We measured inter-brood intervals, egg sizes, brood sizes and brood survival.

## Results

**Figure 1:**
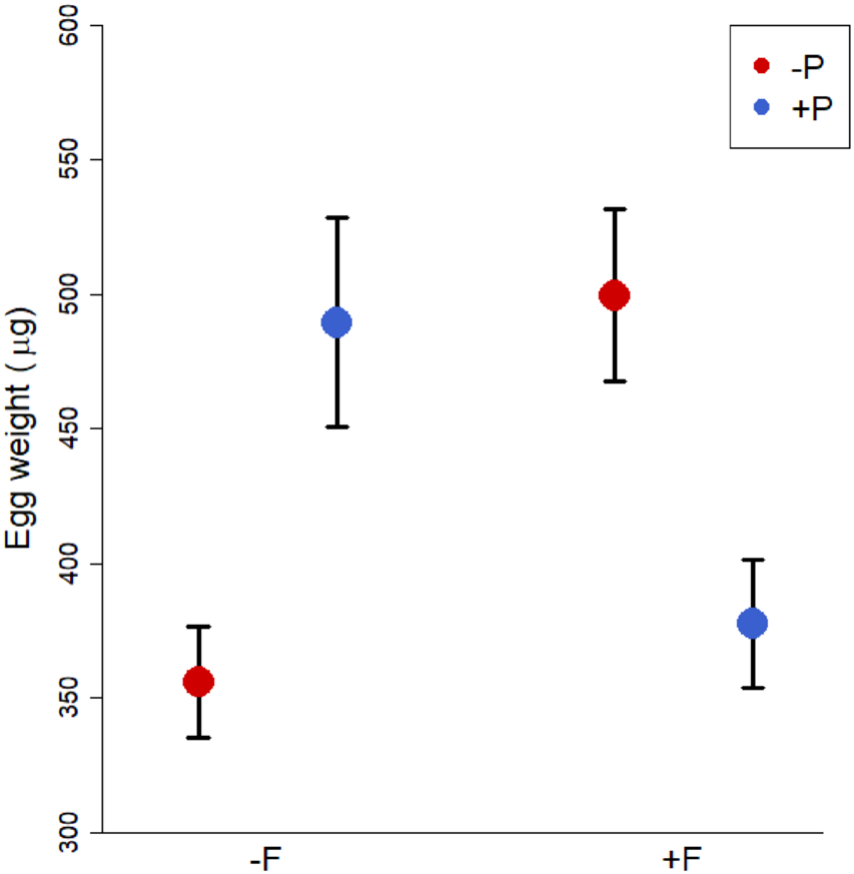
Mean weight per egg (μg) of the first clutch laid by females reared with (+F) or without (-F) older group members that were exposed to predator experience (+P treatment, blue) or no predator experience (−P treatment, red). Means ± SE are shown.

**Figure 2:**
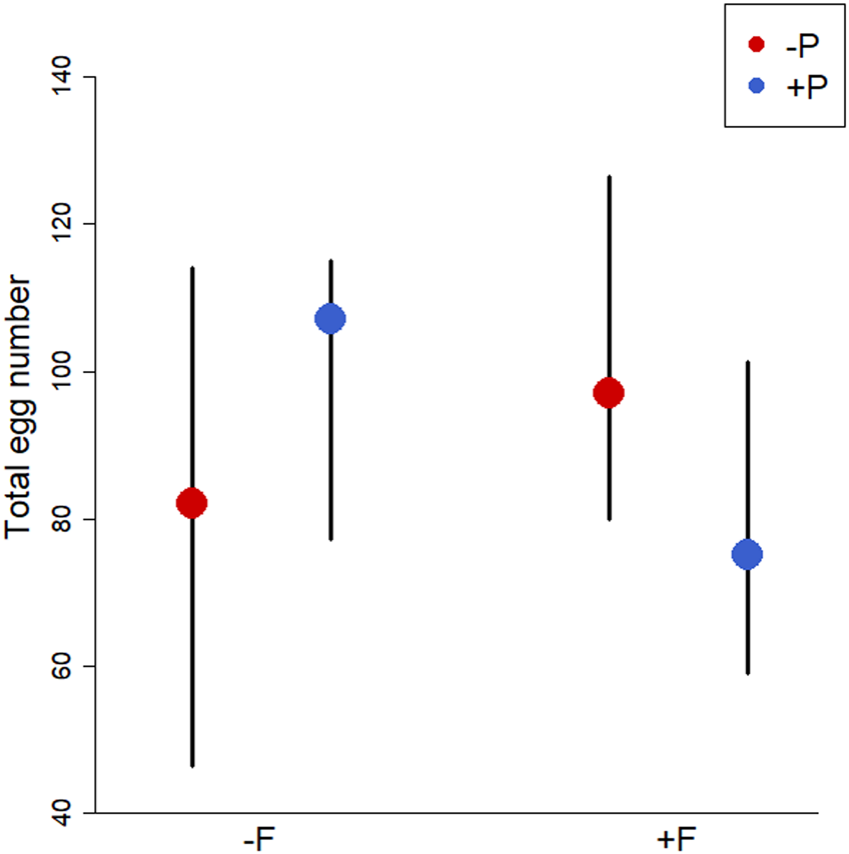
Total egg number produced in the first three clutches by females reared with (+F) or without (−F) older group members that were exposed to predator experience (+P treatment, blue) or no predator experience (−P treatment, red). Medians and interquartile ranges are shown.

There were significant interactions between social and predator early-life treatments on both egg weight (1^st^ clutch; Fig. 1; F × P: p = 0.015, LMM; Table 1a) and total egg number (summed over the first three clutches; Fig. 2; GLMM, F × P: p = 0.0063; Table 2a). Pairs that had been raised with older family members and siblings and with predator experience (+F/ + P) as well as pairs raised with siblings only and without predator experience (−F/−P) laid fewer eggs and smaller eggs than pairs from the other two treatments. Thus, they had a lower reproductive investment compared to pairs raised with siblings only but with predator experience (−F/ + P) and pairs raised with family but without predator experience (+F/−P), which laid larger clutches and bigger eggs (Figs 1,2). Females from the different rearing treatments did not significantly differ in body condition (Table 1b) or in their inter-brood interval (measured between 2^nd^ and 3^rd^ clutch; Table 1c), which means that the lower reproductive investment shown by fish reared under +F/ + P and −F/−P conditions is unlikely to be caused by energetic limitations while producing the three clutches, and it is also not compensated for by a faster rate of clutch production. Offspring survival did not differ between treatments as well (Table 2b).

**Table 1:** Results of linear models to analyse the effect of early social and predator environment on (a) egg weight (LMM, N = 25), (b) female body condition after producing the first clutch (LMM, N = 37) and (c) inter-brood interval between second and third clutch (LM, N = 33).

Factor	Estimate ± SE	Df	t	p
*(a) Egg weight*				
Intercept	373.723 ± 73.072	10.42	5.11	<0.001
Social	127.913 ± 50.803	12.45	2.52	0.026
Predator	125.452 ± 48.158	10.82	2.60	0.025
Egg number	1.322 ± 1.308	18.39	1.01	0.32
Female Weight	−14.242 ± 19.924	17.79	−0.71	0.48
Social × Predator	−226.876 ± 71.009	7.07	−3.19	0.015
*(b) Female body condition*				
Intercept	3.274 ± 0.167	13.93	19.50	<0.001
Social	0.255 ± 0.189	31.54	1.34	0.19
Predator	−0.125 ± 0.189	30.54	−0.66	0.51
*(c) Inter-brood interval*				
Intercept	3.289 ± 0.149	30	21.944	<0.001
Social	−0.161 ± 0.179	30	−0.898	0.376
Predator	−0.130 ± 0.177	30	−0.736	0.467

**Table 2:** Results of GLMMs to analyse the effects of early social and predator environment on (a) clutch size (N = 38) and (b) survival of the second brood (N = 32).

Factor	Estimate ± SE	z	P
*(a) Clutch size*			
Intercept	3.656 ± 0.386	9.48	<0.001
Social	0.367 ± 0.266	1.38	0.17
Predator	0.452 ± 0.263	1.72	0.086
Mean female weight	0.160 ± 0.091	1.74	0.081
Social × Predator	−1.026 ± 0.376	−2.73	0.0063
*(b) Offspring survival*			
Intercept	3.100 ± 0.492	6.29	<0.001
Social	−0.089 ± 0.261	−0.34	0.73
Predator	−0.185 ± 0.272	−0.68	0.50
Age	−0.041 ± 0.012	−3.53	0.00041
Clutch size	0.012 ± 0.008	1.56	0.12

## Discussion

**Figure 3:**
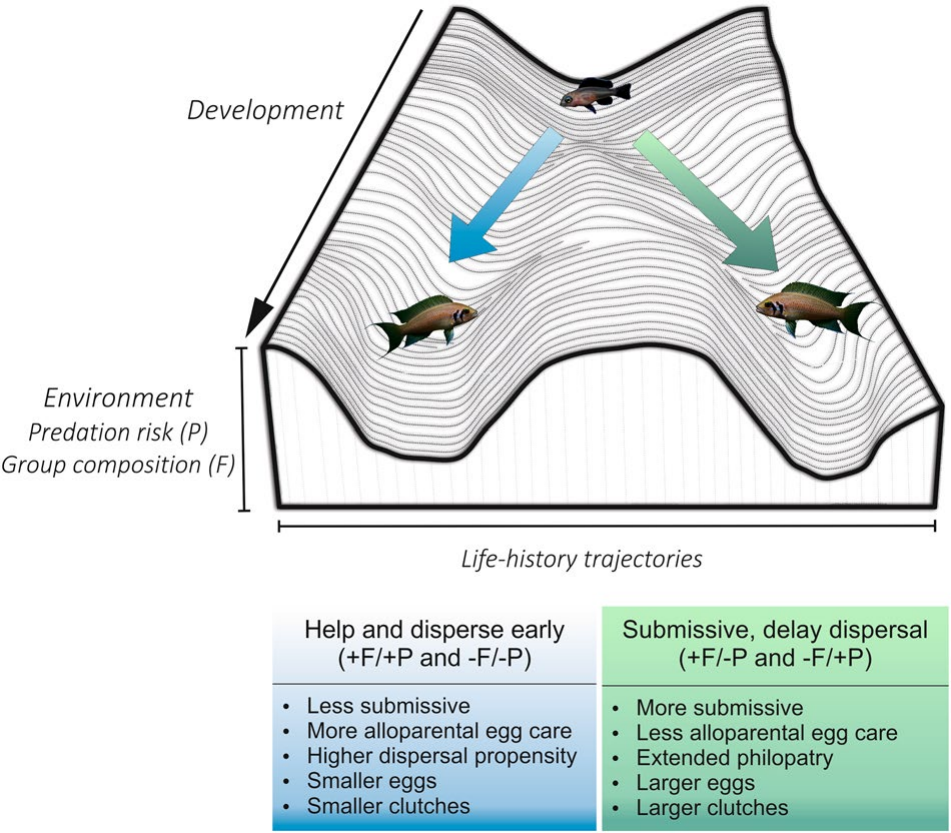
Summary of the alternative life histories of *N. pulcher* based on our experimental results and a previous study using the same experimental population^17^. Here we use Waddington’s landscape metaphor to illustrate how we envisage that starting from a generalist phenotype a specialization into two alternative life history strategies occurs over the developmental time of *N. pulcher*. At the beginning of their development (i.e at the top end of the valley), young *N. pulcher* start life without specialization. The downward slopes of the valleys represents developmental time. The environment in which a young *N. pulcher* is born biases its development to pursue the blue (left valley) or green (right valley) strategy leading to increased specialization with the final outcome of two alternative life-history trajectories. Figure adapted from Waddington’s (1940) landscape^39^.

In 4-yr old *N. pulcher*, social and predation cues experienced in the first two month of life interactively determine which of two life-history trajectories and reproductive strategies are pursued (see Fig. 3 for summary of results). This demonstrates that, like in eusocial insects, cooperative breeders can exhibit environmentally induced lifelong specialization for a reproductive strategy from early life onwards. As several cooperative breeders face the decision between delayed philopatry vs advanced natal dispersal^7–9^, we predict to find phenotypic specialization also in other cooperative systems. For instance, in male Western Bluebirds (*Sialia mexicana*) the combination of aggressive behaviour and dispersal vs. docile behaviour and philopatry are co-selected^8,18^. While in this case behaviour and dispersal tendency seem to be genetically linked, in *N. pulcher* and most other cooperative animal societies social and reproductive roles are non-genetically determined, for instance, by the type of food received (e.g., in honey bees, *Apis melifera*^5,6^), genomic imprinting (e.g., the termite *Reticulitermes speratus*^7^*)*, or, like in some vertebrates, by an age polyethism^10,11^.

In contrast to eusocial systems, individuals pursuing the alternative reproductive strategies reported here in the cooperatively-breeding *N. pulcher* produce fertile offspring, but they do so at a different rate: +F/−P and −F/ + P produced more offspring than +F/ + P and −F/−P fish in their first three broods, whereas inter-brood intervals did not differ. We are confident that the reproductive decisions are a direct consequence of early-life experience rather than cumulative effects of different life experiences during later life, because after the 2-month rearing phase all fish were kept under the same conditions until the breeding trials. In the earlier study that used the same population of experimental *N. pulcher*^17^, different sibling individuals of the rearing groups were used for each behavioural test, so that the results in this study cannot be confounded by experiences individuals made in the previous study. As predicted from the behavioural differences induced by the rearing treatments^17^, early social and predator experience interactively influenced reproduction with fully crossing reaction norms, indicating that behavioural type and reproductive strategy were determined by the same early-life environmental stimuli.

We had hypothesised that early dispersers prepare for taking over a breeder position and should therefore invest more in reproduction than philopatric individuals. However, we found the opposite. Our hypothesis was based merely on our knowledge of the differences in dispersal tendencies between rearing backgrounds. To understand why the results deviate from prediction, instead we should consider how offspring might have perceived their environment *during* the time when the divergence of life-history trajectories was induced, that is, during the first two months of life. The cues we manipulated are related to the social environment and to predation risk. Compared to –F, the presence of breeders and helpers in the +F condition should have provided early-life cues of a higher local density and thus of higher future local competition, but also cues of higher current safety, because these older fish guard young and defend them against predators. Compared to –P, in the +P condition, young perceived cues of current risk, but as predators prey on conspecifics, +P fish may also have perceived cues of reduced future competition. It has been repeatedly shown that young animals sample the environment during early development, which results in phenotypic adjustments to either the current or the envisaged future environment^19^. This, in turn, may lead into specialization of distinct life-history trajectories, as shown in this study. Thus, we have to explain how the combination of cues perceived during early life might have determined the different life-history trajectories.

−F/−P individuals obtained very little information regarding their current or future environment, neither about current predation risk nor on local competition, making their current environment highly uncertain. For instance, the presence of parents during early development helped offspring to learn about the danger posed by heterospecific fish^20^, and the presence of older group members should provide offspring with cues about density and future local competition, because group sizes are autocorrelated across years^21^. –F/−P fish were lacking these cues in early life, and although this is speculative, we hypothesize that these fish developed into dispersers to evade a highly uncertain environment. Conversely, +F/ + P individuals obtained cues of both high local density (presence of breeders and helpers, as compared to –F conditions), and of high predation risk. Thus, +F/ + P might pursue a disperser life history to evade both high predation and high future competition. High population density and within group competition led to decreased reproductive output in some bird and mammalian species^22,23^. In *N. pulcher*, a dispersal trajectory is apparently coupled with reduced reproductive investment. A potential physiological mechanism leading to reduced reproductive investment might be related to enhanced stress levels, that could result from cues of higher density and predation risk (+F/ + P), or from environmental uncertainty (−F/−P). For instance in snowshoe hares (*Lepus americanus*), chronic stress induced by high predation risk negatively affected reproduction^24^. In line with that, *N. pulcher* when raised without parents and predator cues (−F/−P) had a lower expression of the glucocorticoid receptor (GR) gene in the telencephalon^25,26^, which is indicative of a reduced ability to shut down stress responses^27,28^ (stress gene expression in *N. pulcher* reared in +P conditions has not been studied).

−F/ + P individuals experienced cues of a dangerous environment (+P) but no cues of higher population density (−F). Moreover, the predator cues may have indicated reduced future competition levels. Thus, we hypothesize that –F/ + P fish might have developed a philopatric life-history, despite cues of predation risk, because they envisage low levels of future competition and therefore raised chances of territory inheritance. Conversely, +F/−P fish perceived cues of higher future competition, but they grew up in the safest possible of all four rearing treatments: there were no predator cues and, in addition, guarding parents and helpers were present during rearing. Thus, +F/−P rearing conditions represent ‘safe havens’, resulting in a philopatric life-history^15^. The lower risk taken by philopatric individuals might result in lower stress levels compared to disperser fish. Accordingly, +F/−P fish have a higher expression of GR gene in the telencephalon^25,26^, and these fish should therefore be able to shut-off of stress responses faster^27,28^. A more efficient shut-down of stress responses may be part of physiological adaptations leading to higher reproductive investment in philopatric fish. However, current evidence of the relationship between stress hormones and reproductive investment is confined to investigations of basal and peak stress levels, rather than stress response shut-off, and for those parameters evidence was found to be highly context dependent^29^.

Our findings are corroborated by field surveys showing that dispersers have shorter tenure, lower average fitness and lower reproductive success per breeding event when compared with philopatric individuals (Jungwirth *et al*. MS). Openings for breeder positions in the natal territory are fiercely competed for in the field. Thus, individuals of lower competitive ability, which are not able to inherit a tenure position in their home territory, may acquire direct fitness benefits through breeding elsewhere after dispersal, even if dispersing yields lower fitness. Our results suggest that life history decisions occurring during early development might contribute to the reported fitness differences between dispersers and philopatric fish. Alternatively, individuals following a disperser trajectory might lay fewer eggs, because they envisage ending up in smaller groups after dispersal as compared to philopatric individuals. This alternative explanation is also supported by evidence from the field: if subordinates disperse, they end up in smaller groups (Jungwirth *et al*. MS). Under this scenario, dispersers laying smaller clutches could compensate for a lack of help when raising own offspring. However, in that case dispersers should rather lay larger eggs to provide their offspring with a head start, as it was shown in a previous study in *N. pulcher* with experimentally manipulated group sizes^30^.

In conclusion, we showed that a cooperatively breeding species, the cichlid *N. pulcher*, specializes from early life onwards in a high or low reproductive investment strategy. Two environmental factors, the social environment and predation risk, interactively determined the choice of the strategy. The differential investment in egg size between the two strategies is likely to have important implications for offspring size and quality and, together with the different investment in egg number, should influence parental fitness.

## Materials and Methods

### Ethical permits

The current study was conducted in accordance with Swiss Animal Welfare law and was approved by the Veterinary Office of the Kanton Bern, under the licence number 74/15.

### Study species

*N. pulcher* is a cooperatively breeding cichlid fish endemic to Lake Tanganyika^31^, living in groups with a size-based hierarchy consisting of a dominant breeder pair and up to 20 subordinate individuals or helpers^6^. *N. pulcher* is a long-lived species. In its natural habitat, it can reach ages of up to 6–8 years with a mode at 2 years for males and 3 years for females (Jungwirth *et al*. MS). Individuals that acquire a territory as dominant breeders are on average 3.5 years old and territory tenure takes only between 0.5 and 1 years (Jungwirth *et al*. in revision). Depending on population of origin, it reaches standard lengths (SL; i.e. the length between the tip of the snout to the end of the caudal peduncle) of up to 5.5–7 cm. Until sexual maturity, which occurs around an age of 1 year and SL of 3.0–3.5 cm, all subordinate group members delay dispersal from their natal groups and act as alloparental brood care helpers. They engage in different helping behaviours including direct brood care of the dominants’ offspring in form of egg cleaning and fanning, territory maintenance, and defence of the group territory and brood against predators and space competitors^10,32–34^. Many subordinates stay at the natal territory long after sexual maturity, whereas others disperse rather soon afterwards^35^. In *N. pulcher*, severe predation risk is assumed to have selected for the species’ sociality^13^, with social group composition driven by variation in predation pressure^36^, social needs, and conflicts of interest^33^.

### Rearing background of experimental fish

The *N. pulcher* used in our experiment had been reared in two early social environments, either (i) with parents, one helper and same-aged siblings (+F treatment), or (ii) with same-aged siblings only (−F treatment). To manipulate predator experience, individuals of the piscivorous cichlid *Lepidiolamprologus elongatus*, the most dangerous natural predator of *N. pulcher*, were presented for 30 min twice a week to half of the rearing groups (visual and olfactory cues; +P treatment), whereas the other half of the rearing groups received control cues (empty tank and tap water; −P treatment; full details on the rearing procedures are given in Fischer *et al*. 2017^17^). This full factorial rearing design resulted in four treatment combinations (+F/ + P, + F/–P, –F/ + P and –F/–P). The early-environment treatments were applied for 62 days after larvae had reached the free-swimming stage, which occurs at 10 days of age^17^. Subsequently to the early-environment treatments, fish from all treatments were kept separated by sex in same-age sibling groups within 200 L tanks.

### Husbandry of experimental fish

The experiment was conducted at the ‘Ethologische Station Hasli’ of the Institute of Ecology and Evolution, University of Bern, Switzerland, under licence number 74/15 of the Veterinary Office of the Kanton Bern. Experimental pairs were kept in 50-L compartments of 200-L tanks. Each 50-L compartment was equipped with a 2-cm sand layer, a biological filter and two clay flower pot halves as shelters. The light:dark cycle was set to 13:11 h with a 10 min dimmed light period in the morning and evening to simulate the light conditions of Lake Tanganyika. Fish were fed *ad libitum* with commercial flake food TetraMin at 5 days a week and frozen zooplankton at one day a week. Water temperature was maintained at 27 ± 1 °C.

### Experimental breeding trials

When the experimental *N. pulcher* were 4 years old, we formed ten breeding pairs of fish from each of the four rearing treatments (in total 40 pairs). In their natural environment, at this age surviving males and females would have reached a breeder position in a group. We separated ten 200-L tanks into four 50-L compartments each, resulting in a total of 40 watertight compartments, separated by opaque PVC dividers sealed by silicon seams, prohibiting any olfactory and visual contact between compartments. The 40 breeding pairs were distributed over the 40 compartments such that in each of the four compartments of a 200-L tanks all four treatments were present. Both members of each pair had experienced the same combination of early-life social and predator treatments. Pair members were unrelated and unfamiliar to each other. Females were chosen such that on average their standard length did not differ between the four treatments combinations (ANOVA: + F/ + P: t = −0.532, p = 0.59; −F/ + P: t = −1.542, p = 0.13; −F/−P: t = −0.160, p = 0.87; N = 10 for all treatments). Males were chosen to be 10 mm standard length (SL) larger than their female partner to mimic the size differences found in natural pairs. Male length also did not differ between treatments (ANOVA: + F/ + P: t = 0.144, p = 0.88; −F/ + P: t = −1.435, p = 0.16; −F/−P: t = 0.191, p = 0.84; N = 10 for all treatments). All pairs were checked twice a day over the first three days after co-housing to assess if a pairing was successful or not. A pairing was rated as ‘unsuccessful’ if within these first three days one pair partner was evicted, i.e., if it stayed close to the water surface most of the time or received aggression by the other fish repeatedly. As soon as we observed first signs of eviction, we immediately separated the two fish and re-paired both individuals with another prospective partner.

### Reproductive performance

Each pair was checked daily over a period of 6 months for the presence of clutches, or until three clutches had been produced. Within one day of spawning of the first clutch, we recorded egg number and female weight and subsequently obtained the dry weight of the eggs by drying the entire clutch at 60 °C for 12 h. The dry weight per egg, further referred to as ‘egg weight’, was determined as clutch dry weight, weighed to the nearest 0.01 mg, divided by egg number. Further we recorded laying date, egg number and female weight within one day of the spawning of second and third clutches. Because we removed all first clutches of the pairs, the interval between first and second clutch is likely to be shorter than in undisturbed pairs. This is because a pair will lay a consecutive clutch faster if it did not have to spend time and energy for parental care. Therefore, we used the interval between 2^nd^ and 3^rd^ clutch, when fish were left undisturbed, as a measure of inter-brood intervals. We calculated Fulton’s body condition index for females after laying the first clutch as F = mass (g)/standard length (cm^3^) × 100. To obtain a measure of offspring survival we counted the number of free-swimming fry, produced in the second clutch within 2 days after the pair laid the third clutch and corrected for offspring age in the statistical models (see next section).

### Statistical Analysis

Statistical analyses were performed by fitting mixed models using the software R version 3.5.2^37^. In all models we included the identity of the females’ parents of our experimental pairs as random factor to account for genetic background. Furthermore, all models contained the social (F) and predator (P) rearing treatments as fixed effects. All initial models also contained the F × P interaction term. This term was dropped by backwards elimination if it was not significant^38^. We analysed egg weights of 1^st^ clutches and female body condition after laying the first clutch with linear mixed-effect models (LMMs) using the package lme4^38^. The assumptions of normality of the error term were checked by Shapiro-Wilk tests, and visual inspection of quantile-quantile plot of model residuals to test for skew and kurtosis, as well as plots of residuals vs. fitted values to test for homogeneity of variance. In the model for egg weight, we included clutch size and female body weight as additional fixed factors. We analysed the egg weights of a total of 25 clutches which were distributed over the four treatment as follows: seven clutches for +F/ + P, five for +F/−P, eight for−F/ + P and five for the −F/−P treatment. Egg weights of 15 clutches are lacking, because we did not find the eggs before hatching. In these 15 cases we counted the number of fry (10 days old) to obtain an estimate of clutch size. The interval between the second and third clutch was log transformed to fit a normal distribution and analysed with an LMM. The normality assumptions were checked as described above. In this model, the identity of female’s parents, which was initially included in all models (see above), explained zero variance of the data, which is why in this case we fitted a linear model without this random factor. The total number of eggs produced in the first three consecutive clutches was analysed by fitting generalized mixed-effects models (GLMMs) with log-link function as the data followed a Poisson distribution. In the GLMM for egg number, mean female weight was included as additional fixed factor, and an observation-level random factor was included to correct for overdispersion. Offspring survival of the second clutch was analysed by fitting a GLMM with log-link function assuming a negative binomial distribution. Offspring age and clutch size were included as additional fixed factors.
